# Targeting the Endocannabinoid System in the Treatment of Posttraumatic Stress Disorder: A Promising Case of Preclinical-Clinical Translation?

**DOI:** 10.1016/j.biopsych.2021.07.019

**Published:** 2021-07-24

**Authors:** Leah M. Mayo, Christine A. Rabinak, Matthew N. Hill, Markus Heilig

**Affiliations:** Center for Social and Affective Neuroscience, Department of Biomedical and Clinical Science, Linköping University, Linköping, Sweden; Department of Pharmacy Practice, Translational Neuroscience Program, Psychiatry and Behavioral Neurosciences, Wayne State University, Detroit, Michigan; Department of Cell Biology and Anatomy & Psychiatry, Hotchkiss Brain Institute and the Mathison Centre for Mental Health Research and Education, University of Calgary, Calgary, Alberta, Canada; Center for Social and Affective Neuroscience, Department of Biomedical and Clinical Science, Linköping University, Linköping, Sweden

## Abstract

The endocannabinoid (eCB) system is one the most ubiquitous signaling systems of the brain and offers a rich pharmacology including multiple druggable targets. Preclinical research shows that eCB activity influences functional connectivity between the prefrontal cortex and amygdala and thereby influences an organism’s ability to cope with threats and stressful experiences. Animal studies show that CB_1_ receptor activation within the amygdala is essential for extinction of fear memories. Failure to extinguish traumatic memories is a core symptom of posttraumatic stress disorder, suggesting that potentiating eCB signaling may have a therapeutic potential in this condition. However, it has been unknown whether animal findings in this domain translate to humans. Data to inform this critical question are now emerging and are the focus of this review.

We first briefly summarize the biology of the eCB system and the animal studies that support its role in fear extinction and stress responding. We then discuss the pharmacological eCB-targeting strategies that may be exploited for therapeutic purposes: direct CB_1_ receptor activation, using Δ^9^-tetrahydrocannabinol or its synthetic analogs; or indirect potentiation, through inhibition of eCB-degrading enzymes, the anandamide-degrading enzyme fatty acid amide hydrolase; or the 2-AG (2-arachidonoyl glycerol)–degrading enzyme monoacylglycerol lipase. We then review recent human data on direct CB_1_ receptor activation via Δ^9^-tetrahydrocannabinol and anandamide potentiation through fatty acid amide hydrolase blockade. The available human data consistently support a translation of animal findings on fear memories and stress reactivity and suggest a potential therapeutic utility in humans.

Posttraumatic stress disorder (PTSD) is a debilitating condition that occurs in a subset of people after exposure to a life-threatening event or threat of serious bodily injury (1). The course of PTSD is chronic and often severe, with many patients taking decades to achieve remission, and remission is frequently incomplete (2,3). Over time, initial avoidance of specific trauma-associated stimuli generalizes to once benign cues and contexts, resulting in progressive withdrawal from family, friends, and work life, which perpetuates impaired functioning. In PTSD, fear extinction deficits contribute to the persistence of traumatic memories. These impairments are associated with decreased activation in the ventromedial prefrontal cortex (vmPFC) and hippocampus, brain regions involved in fear extinction and recall of extinction learning (i.e., extinction retention), as well as increased activation of the amygdala, an area critical to threat responding (4). Although fear extinction is impaired in patients with PTSD (5), standardized cognitive behavioral therapy approaches that facilitate extinction learning can ameliorate these deficits (6).

Interventions to promote extinction learning are the foundation of PTSD treatment, and their efficacy is supported by meta-analyses (7,8). The prototypical example, prolonged exposure (PE) therapy, uses imaginal and in vivo exposures to trauma-associated stimuli to promote extinction learning (9,10). Standard PE treatment consists of weekly sessions conducted over a period of approximately 3 months (1). PE has shown positive results in well-designed, adequately powered, randomized controlled PTSD trials (11,12). While generally effective, current treatments—even when available to patients—are insufficient. First, exposure-based treatment is associated with an increased dropout rate [see e.g., (12)]. Patients have trouble managing distress during treatment when recalling memories of the traumatic experience. Even if treatment is successful and patients no longer meet criteria for a PTSD diagnosis, subthreshold symptoms often persist (13). Although extinction learning establishes a new, safe association, the original fear memory persists, and spontaneous renewal of fear is common (2,8). Finally, heterogeneity within PTSD symptomatology results in a diverse patient population, highlighting the need for biomarkers that provide insight into who may benefit most from a given treatment (14).

Pharmacotherapy could augment psychotherapy in at least two important ways. By potentiating key biological mechanisms that mediate extinction learning, more robust and lasting results could be achieved, perhaps even with more accessible interventions that are less resource demanding, such as internet-based exposure therapy (15). Furthermore, pharmacological interventions could help patients tolerate the distress of trauma-memory exposure without interfering with extinction learning mechanisms. Currently available pharmacotherapies for PTSD do not achieve these objectives (16). First-line pharmacological treatments, such as selective serotonin reuptake inhibitors, mitigate some symptoms of PTSD but fail to engage the core underlying pathophysiology. As a result, these medications are no more effective when used with PE than without (11), and there is thus a need for novel medications to potentiate fear extinction during exposure therapy. The endocannabinoid (eCB) system plays a key role in fear extinction and buffering of stress (17), and several strategies that potentiate eCB signaling (Figure 1) are emerging as candidate treatments for PTSD.

## THE eCB SYSTEM IN FEAR AND STRESS: PRECLINICAL EVIDENCE

### An Introduction to the eCB System

**Figure 1.**
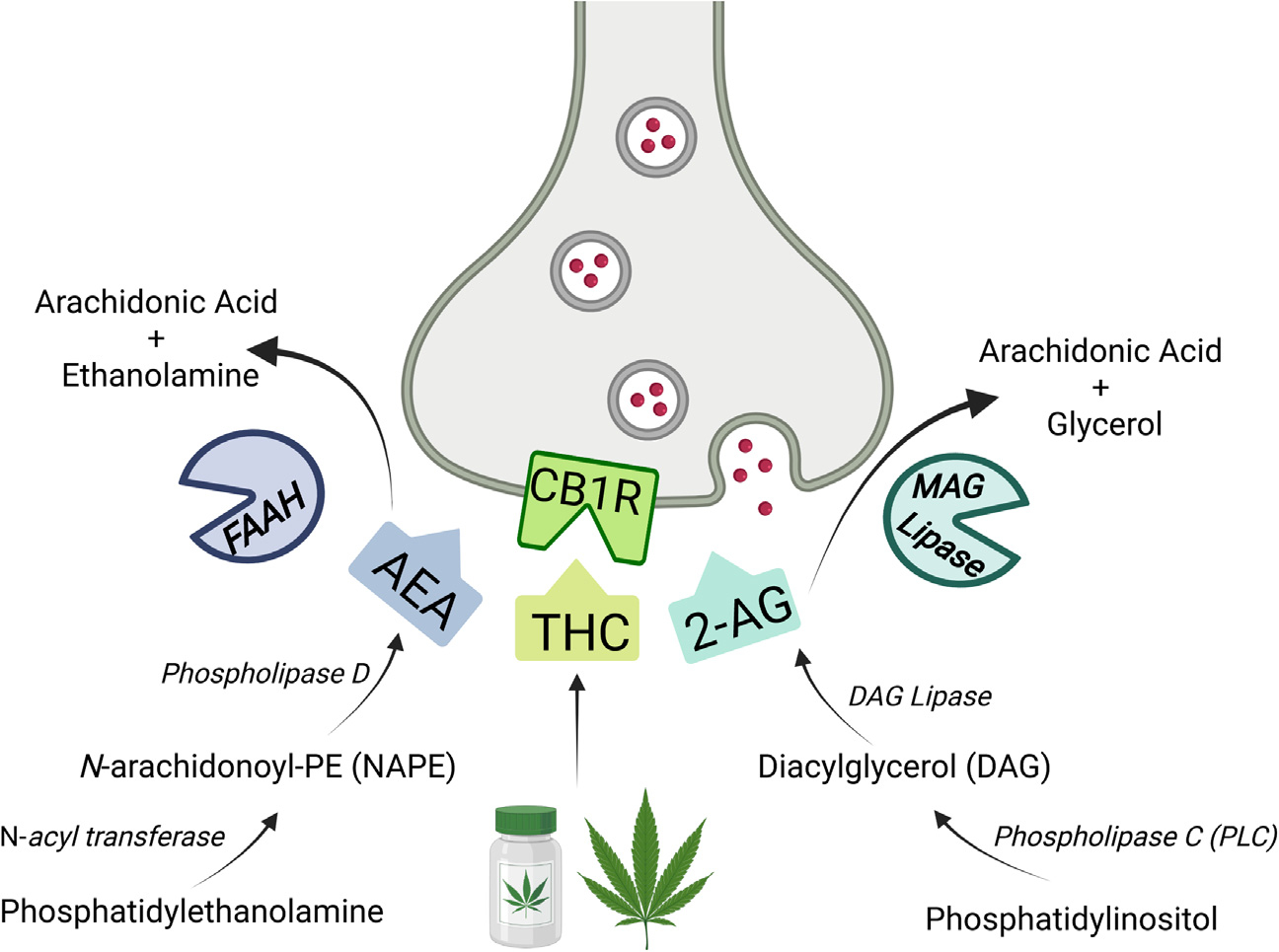
Overview of therapeutic targets to enhance cannabinoid signaling. Currently, there are three distinct ways to modulate cannabinoid signaling via the CB_1_R in humans. The primary psychoactive ingredient of cannabis, THC, can activate CB_1_Rs and has been used in humans extensively for medical and nonmedical purposes. More recently, enzyme inhibition approaches have been used to modulate endogenous cannabinoid function. Inhibition of FAAH can increase AEA availability, while inhibition of MAGL can similarly promote 2-AG. All three methods appear to influence stress and fear learning, although dose and route of administration may be critical. Currently, THC and FAAH inhibition are each being used together with prolonged exposure therapy to treat posttraumatic stress disorder in separate clinical trials. MAGL inhibition is currently being evaluated on neuroimaging biomarkers in posttraumatic stress disorder population, as well. Future work will hopefully highlight the shared and unique features of each pharmacological target in this patient population. Figure created with BioRender.com. 2-AG, 2-arachidonoyl glycerol; AEA, anandamide; CB_1_R, CB_1_ receptor; FAAH, fatty acid amide hydrolase; MAGL, monoacylglycerol lipase; THC, Δ^9^-tetrahydrocannabinol.

The eCB system was first characterized as the biochemical target through which Δ^9^-tetrahydrocannabinol (THC), the psychoactive constituent of cannabis, exerts its effects. The eCB system is neuromodulatory and composed of two CB receptors, type 1 (CB_1_) and type 2 (CB_2_) (18, 19), and two major endogenous ligands, anandamide (AEA) (20) and 2-AG (2-arachidonoyl glycerol) (21). THC and AEA are partial agonists at CB_1_ and CB_2_ receptors, while 2-AG is a full agonist at both receptors. AEA and 2-AG are rapidly synthesized on-demand and act in a retrograde fashion to activate CB_1_ receptors and reduce presynaptic neurotransmitter release. The primary biosynthetic enzyme for AEA is NAPE-PLD, although there are multiple redundant pathways for AEA synthesis (22). Degradation of AEA, however, is almost exclusively mediated by fatty acid amide hydrolase (FAAH) (22,23). 2-AG biosynthesis is mediated by the conversion of diacylglycerol to 2-AG by the enzyme diacylglycerol lipase, while its degradation is largely by monoacylglycerol lipase (MAGL) (22,24) (Figure 1).

### Regulation of Fear and Stress by CB_1_ Receptor Agonists

#### Fear.

The impact of CB_1_ receptor agonists on fear is dependent on dose, route of administration, and the phase of fear learning during which drug is administered; detailed reviews of this work can be found elsewhere (17,25). The effects of CB_1_ receptor agonists specifically on fear extinction are much more consistent. Administration of CB_1_ receptor agonists, systemically or locally within the amygdala, PFC, or hippocampus, reliably enhances fear extinction, particularly the consolidation of extinction (26–32). CB_1_ receptor agonists can also disrupt the reconsolidation of fear memories through their actions in the amygdala, hippocampus, and several cortical regions (26,31,33). Together, these actions suggest that CB_1_ receptor agonists can both suppress established fear memories and facilitate their extinction.

#### Stress.

Systemic administration of CB_1_ receptor agonists exhibits biphasic effects on stress responses, whereby lower doses often attenuate various hormonal and behavioral effects of stress, while higher doses amplify them (30,34,35). The basolateral amygdala (BLA) is a critical site of action for the stress-reducing effects of CB_1_ receptor activation, as local administration of CB_1_ receptor agonists into the BLA suppresses stress-induced corticosterone secretion and impairments in fear extinction (36–38). The neural circuits or cell types through which high-dose CB_1_ receptor agonists exert their amplifying effects on the stress response are not well characterized, but potentially involve actions at alternate amygdala nuclei (36,39). Stress-reducing effects of CB_1_ receptor activation may involve CB_1_ receptors on glutamatergic neurons, while the stress-amplifying effects of high-dose cannabinoids may be mediated by CB_1_ receptors on GABAergic (gamma-aminobutyric acidergic) neurons (40,41).

### Regulation of Fear and Stress by FAAH Inhibitors

#### Fear.

Effects of FAAH inhibitors on fear and stress largely parallel those seen with CB_1_ receptor agonists, but FAAH inhibitors do not typically exhibit biphasic effects. Endogenous AEA signaling promotes fear learning, and amplifying AEA via inhibition of FAAH enhances fear learning. Elevating AEA signaling locally within the PFC, amygdala, and hippocampus during fear learning can facilitate consolidation of fear memories (42–44).

Acute inhibition of FAAH or AEA uptake prior to fear extinction can increase extinction, with evidence for both enhancement of within-session extinction and consolidation of extinction. Repeated administration of a FAAH inhibitor during exposure to reminder cues or extinction training also enhances long-term consolidation of extinction memories (45). Similarly, acute FAAH inhibition can suppress the expression of learned fear (46) and reduce escape-like behavior in response to an artificial predator (47). Enhanced fear extinction is also seen in mice with a loss-of-function *FAAH* mutation that results in elevated AEA signaling (48). In fact, elevations in AEA signaling can mediate the pro-extinction effects produced by other drugs, including cannabidiol (49) and fluoxetine (50). Conversely, AEA depletion by global inhibition of its biosynthetic enzyme NAPE-PLD (51) or selective overexpression of FAAH within the hippocampus (52) impairs fear extinction, suggesting that AEA signaling is necessary for this process. Collectively, these data create a compelling argument that AEA signaling is involved in fear extinction and that its elevation via inhibition of FAAH is capable of augmenting fear extinction, particularly its consolidation.

#### Stress.

AEA signaling largely acts to constrain the stress response. Central AEA depletion via inhibition of NAPE-PLD rapidly activates the hypothalamic-pituitary-adrenal (HPA) axis and promotes release of corticosterone (51). The amygdala is a primary hub through which AEA signaling constrains activation of stress responses (40). Under neutral conditions, a tonic AEA signal is active at synapses within the BLA, where it limits glutamate release (53). Exposure to stress drives the release of corticotropin-releasing factor and activates CRF_1_ receptors, causing a rapid induction of FAAH activity (54). This stress-induced increase in FAAH depletes the signaling pool of AEA within the BLA and disinhibits glutamatergic neurotransmission (40). Inhibition of FAAH can prevent stress-induced increases in glutamate release in the BLA (53), and local blockade of FAAH within the BLA can blunt stress-induced activation of the HPA axis, anxiety-like behavior, and fear extinction impairments (55,56). Together, these data indicate that stress-induced release of corticotropin-releasing factor triggers rapid elevations in FAAH activity and reductions in AEA signaling and that these contribute to generating a stress response.

### Regulation of Fear and Stress by MAGL Inhibitors

#### Fear.

Pharmacological and genetic tools to manipulate 2-AG biosynthesis and hydrolysis have emerged more recently (57). Thus, the impact of 2-AG signaling on fear learning and extinction is not as well characterized as that for AEA. For example, inhibition of MAGL prior to fear learning dampens fear responses during acquisition (44), but inhibition of MAGL after learning can increase consolidation of fear memory (58). Contrary to FAAH inhibition, MAGL inhibition enhances fear expression (46). Similarly, MAGL inhibition enhances defensive flight responses to a naturalistic predator, which is opposite to FAAH inhibition (47). Also in direct opposition to the effects of FAAH inhibition, pharmacological inactivation of MAGL impairs short-term fear extinction (59) and has minimal impact on consolidation of extinction (45). Interestingly, genetic (60) or pharmacological (61) inhibition of 2-AG biosynthesis by the diacylglycerol lipase enzyme also results in an impairment of fear extinction. Collectively, 2-AG signaling may promote fear learning similar to other CB_1_ receptor agonists, while its role in extinction is more complex. Although 2-AG signaling is requisite for fear extinction, elevating 2-AG signaling robustly impairs fear extinction. The most parsimonious explanation is that endogenous 2-AG acts at CB_1_ receptors on glutamate neurons to govern fear extinction, but if 2-AG signaling is dramatically elevated, it activates CB_1_ receptors on GABAergic neurons to enhance fear. This biphasic model is consistent with what is known about CB_1_ regulation of anxiety (62); however, it is unclear why these biphasic actions are seen with exogenous CB_1_ receptor agonists and elevations in 2-AG signaling but not AEA signaling.

#### Stress.

The regulation of stress-related processes by 2-AG signaling is similarly equivocal. For instance, glucocorticoid hormones released in response to stress mobilize 2-AG signaling (63–65). This dynamic change in 2-AG signaling, however, seems to both dampen some aspects of the stress response, such as reducing corticosterone responses to stress (66,67), and promote some adverse effects of stress, such as elevations in anxiety (68) and drug seeking (69). As mentioned above, several of these effects appear to be attributable to actions of 2-AG signaling at CB_1_ receptors on GABAergic neurons (66,68,69). However, the influence of 2-AG signaling on HPA axis dynamics are not consistent, as MAGL inhibition has been found to also increase basal corticosterone (70), potentiate HPA axis activation during a social challenge (71), and prolong corticosterone secretion after exposure to stress, resulting in impaired termination of HPA axis activity (67). Therefore, the impact of 2-AG signaling on stress-induced regulation of the HPA axis is currently unclear.

Examination of stress-induced anxiety shows a more consistent and encouraging picture. MAGL inhibition reliably reduces acute anxiety states on stress exposure (72–74), reduces anxiety states that develop over a sustained period after exposure to stress (75,76), and promotes resilience against the development of persistent anxiety after exposure to repeated stress (77).

Collectively, these data create a complex picture regarding the role of 2-AG in regulation of fear and stress responses. Physiologically, 2-AG signaling may be involved in fear extinction, but pharmacological MAGL inhibition can enhance the expression of conditioned fear and impair its extinction. This indicates that supraphysiological elevations in 2-AG signaling enhance fear rather than suppress it. With respect to stress, it is unclear how 2-AG signaling affects HPA axis activity, as studies have reported both dampening and activating effects. Data more consistently indicate that MAGL inhibition suppresses stress-induced anxiety across a multitude of exposure durations and models.

The preclinical findings reviewed above have some clear implications for development of clinical PTSD treatments:

1. FAAH inhibitors currently have the most consistent rationale as candidate PTSD treatments. Preclinical research consistently indicates that FAAH inhibition can enhance fear extinction and consolidation and suppress multiple aspects of the stress response, particularly, changes in anxiety and neuroendocrine function.
2. Exogenous direct CB_1_ receptor agonists have some promise for the regulation of fear and stress, as they also enhance fear extinction and dampen stress responses. Notably, these effects are highly dependent on dose and may still carry the risk of effects on other CB_1_-mediated functions, such as appetite, cognition, and motor coordination.
3. MAGL inhibitors may have some potential to mitigate stress-induced anxiety, but consistent evidence supporting their utility as PTSD treatments is presently lacking.

## THE eCB SYSTEM IN FEAR AND STRESS: EVIDENCE FROM HUMAN STUDIES

Cross-sectional evidence from clinical populations supports the notion that the eCB system is dysregulated in PTSD (78,79). Patients with PTSD have lower peripheral 2-AG levels overall, while decreased AEA levels correlate with the number and severity of intrusive symptoms. However, altered eCB levels may represent a vulnerability factor for PTSD development rather than a consequence of the disorder. Experimental studies are thus critical for assessing the direction and causal relationship between eCB levels and PTSD symptoms, and the use of AEA and 2-AG levels as biomarkers of in vivo FAAH and MAGL inhibitor activity, respectively, may be a key asset for clinical development efforts.

### Experimental Evidence: THC

#### Fear.

The role of cannabinoids in fear extinction has recently been characterized in humans. In healthy adults, an acute low dose of THC administered prior to extinction learning enhanced recall of the extinction memory. THC did not affect within-session fear extinction but influenced the ability to successfully recall extinction learning, suggesting that THC affects the ability to maintain and/or successfully retrieve extinction memories [(80), but see (81)]. In subsequent studies, THC administration attenuated amygdala reactivity during early extinction, as well as increased vmPFC and hippocampal activation (82) and increased functional connectivity between the vmPFC and hippocampus (83) during an extinction memory recall test 24 hours and 1 week after extinction learning, respectively. THC-induced changes in neural activity during fear extinction were sustained after extinction learning, potentially reflecting ongoing processes involved in consolidation of the extinction memory (84). Together, these data connect mechanistic insights from rodent studies of fear extinction to human neuropsychopharmacology, providing initial proof of concept for potential clinical utility of targeting cannabinoid receptors in the treatment of PTSD.

#### Stress.

Studies suggest that cannabis is commonly used for stress relief and relaxation (85). In line with this, a controlled laboratory study in healthy adults found that a low dose of THC (7.5 mg) reduced the negative emotional effects of a standardized psychosocial stressor without impacting performance. In contrast, and consistent with preclinical data discussed above, a higher dose of THC (12.5 mg) increased negative affect and subjective distress (86).

### Experimental Evidence: FAAH Inhibition

Initial human evidence supporting the therapeutic potential of FAAH inhibition comes from behavioral genetic studies. A *FAAH* 385C→A loss-of-function mutation encodes a FAAH protein that is degraded more rapidly, resulting in reduced FAAH activity and elevated peripheral AEA levels (48,87–89). Using [^11^C]CURB, a positron emission tomography–ligand for FAAH, it has been shown that brain FAAH protein levels are lower in *FAAH* 385A carriers, providing indirect evidence that levels of AEA in these individuals are likely to be elevated because of reduced peripheral and central FAAH levels (90). In healthy individuals, lower levels of FAAH expression in the amygdala correlate with greater functional connectivity of the vmPFC and the amygdala—a neural signature of enhanced fear extinction (91).

#### Fear.

An innovative translational approach has provided convergent biochemical, behavioral, and neural data demonstrating beneficial effects of elevated AEA resulting from reduced FAAH activity (48). The loss-of-function *FAAH* 385A allele was inserted into mice, resulting in decreased FAAH activity and concomitantly increased AEA levels. In mice and humans, carriers of the A-allele had enhanced fronto-amygdala connectivity and fear extinction. We subsequently reported similar effects in humans, including a gene dose–dependent effect of the A-allele on peripheral AEA levels (87). Individuals homozygous for the A-allele showed enhanced fear extinction and recall of extinction learning when tested 24 hours later. These findings have since been replicated by others (92), showing that fear extinction is related to peripheral AEA levels (93,94). Collectively, the human genetic results consistently support a translational validity of preclinical findings on the role of AEA in consolidation of fear extinction memory (95) and provide initial proof of principle for FAAH inhibition as a mechanism to enhance fear extinction. However, genetic studies cannot distinguish between developmental and direct functional consequences of genetic variation (96). This question, which is critical to determine the therapeutic potential of FAAH inhibition, can only be resolved by interventional studies.

To date, only two studies have assessed the effects of pharmacological FAAH inhibition on fear learning (97,98). In healthy adults, the effects of FAAH inhibition were evaluated using PF-04457845, a covalent (irreversible) FAAH inhibitor that is orally available, brain penetrant, and able to sustain a complete or near-complete inhibition of FAAH activity for 24 hours on administration once daily (99,100). PF-04457845, administered once daily for 10 days at a dose of 4 mg, was well tolerated and produced enhanced recall of extinction memory when tested 24 hours after extinction learning (97). In contrast, PF-04457845 did not influence the acquisition of fear conditioning or within-session extinction. The inability of PF-0445785 to facilitate within-session extinction may seem unexpected, because elevated AEA levels in *FAAH* 385A carriers were associated with enhanced extinction (48,87). The other FAAH inhibitor tested for its effects on conditioned fear, JNJ-42165279 (101), also failed to facilitate within-session extinction (98). In the latter study, the authors used a 1-day conditioning paradigm that did not test extinction memory recall after overnight consolidation. It is therefore currently unknown whether JNJ-42165279 is able to promote extinction consolidation.

In summary, reduced FAAH activity, whether due to genetic variation or pharmacological inhibition, appears to reliably promote recall of fear extinction, in accordance with preclinical findings (95). Enhanced prefrontal control over the amygdala, a critical component of extinction memory formation, offers a candidate mechanism to mediate this action (48,102). Findings are less consistent regarding within-session extinction, with discrepancies between consequences of elevated AEA conferred via *FAAH C385A* variation and pharmacological FAAH inhibition (48,87,97,98,103). This discrepancy may suggest that while enhanced consolidation of extinction memory is a direct functional consequence of elevated AEA levels, enhanced within-session extinction in carriers of loss-of-function allele may represent a developmental effect. In addition, sex/gender differences exist in both PTSD development (104) and endocannabinoid function (103) but have not been addressed in all studies (Table 1).

#### Stress.

The stress-buffering effects of AEA demonstrated in preclinical animal models are supported by initial behavioral genetic studies in humans. Although not entirely consistent across samples, healthy humans exposed to an acute laboratory stressor show a transient increase in peripheral AEA levels [(87,105,106), but see (107)], followed by a subsequent decrease during recovery (87,108). Humans and mice homozygous for the *FAAH* 385 A-allele have elevated AEA at baseline and fail to show decreases in AEA levels during stress recovery (87). In humans, decreased AEA during stress recovery coincides with enhanced negative affect, as indexed via facial electromyography. Individuals homozygous for the Aallele are impervious to stress-induced decreases in AEA and related increases in negative emotion (87), suggesting that AEA buffers the negative emotional consequences of stress (25).

There is also evidence of stress-buffering effects of AEA in clinical populations. Individuals with PTSD and comorbid alcohol use disorder who carry the variant A-allele also have higher peripheral AEA levels (89). Moreover, they self-report less anxiety in response to a stress script and show greater improvements on the PTSD symptom of hyperarousal. Thus, elevating AEA may offer a pharmacological mechanism for treatment of anxiety-related disorders, particularly in patient populations characterized by elevated levels of arousal. However, it should be noted that increased stress load, including exposure to trauma during childhood (109) or genetically conferred hyper-reactivity of the HPA axis (110), may render A-allele carriers more vulnerable to developing anxiety-related disorders. Moreover, translational evidence pinpoints a critical sensitive period of endocannabinoid development during adolescence (96), suggesting that developmental timing of cannabinoid interventions may be particularly critical.

Thus far, only one study has explored the pharmacological effects of FAAH inhibition on stress responses (97). In the PF-04457845 study reviewed above, 10 days of FAAH inhibition produced no detectable effects on baseline mood or anxiety. However, on exposure to a laboratory stress challenge, individuals receiving the inhibitor had attenuated subjective and psychophysiological responses as well as less negative emotionality. These effects are similar to, but broader than, those obtained in genetic elevation of AEA, where only reduced negative emotionality after exposure to the laboratory stressor was found (87). A likely reason for the difference between these observations is that the magnitude of AEA elevation produced by genetic variation, approximately 25%, is modest compared with the nearly 10-fold elevation seen after pharmacological FAAH inhibition. Thus, AEA elevations resulting from pharmacological FAAH inhibition may produce stress-buffering effects that go beyond those observed as a consequence of FAAH C385A genetic variation.

### Experimental Evidence: MAGL Inhibition

A positron emission tomography study has been completed to evaluate central MAGL occupancy by the inhibitor Lu AG06446 (NCT04419636), but results have yet to be published.

## CLINICAL TRIALS

Deficits in retention of fear extinction memory have been observed in PTSD populations, while multiple lines of evidence reviewed above support the ability of direct CB_1_ activation or enhanced eCB transmission to promote extinction recall. The cannabinoid system therefore seems to offer opportunities for developing novel PTSD treatments, either by acting on their own or by improving exposure-based therapies that are aimed at extinguishing traumatic memories. Potential benefits in the latter case may include improving the tolerability of exposure-based treatments, resulting in lower dropout rates, and potentially allowing treatment to be shortened while maintaining or improving its efficacy.

### Clinical Trials: THC

A recent clinical trial found no effect of ad libitum smoked cannabis on PTSD symptoms over a 3-week period (111). However, two clinical trials with controlled dosing currently underway may provide a more mechanistic insight, specifically testing the hypothesis that direct activation of CB_1_ receptors through administration of THC will enhance recall of fear extinction in patients with PTSD (NCT02069366; NCT03008005). Another ongoing clinical trial is testing the ability of THC to increase the effectiveness of exposure-based therapy in PTSD (NCT04080427). Data from a preliminary study found that THC modulated threat-related processing in individuals with PTSD (112), further supporting the notion that THC may prove advantageous as a pharmacological approach to treating trauma-related psychopathology, particularly when administered together with psychotherapy.

### Clinical Trials: FAAH Inhibition

The FAAH inhibitor PF-04457845 was originally developed for the treatment of osteoarthritic pain but only showed modest efficacy and was thus terminated in development for this indication (113). Following preclinical findings supporting a role of FAAH inhibition in fear- and stress-related behaviors, two studies were started in 2014. One was to evaluate the ability of PF-04457845 to attenuate negative emotional responses and facilitate fear extinction in patients with PTSD using a neuroimaging paradigm (NCT02216097). The other study was to evaluate the efficacy of PF-04457845 in combination with PE-based therapy in patients with PTSD and comorbid alcohol use disorder (EudraCT 2014–002456-90). These two, and all other trials with FAAH inhibitors, were abruptly halted when administration of a novel FAAH inhibitor, BIA 10–2474, resulted in a fatality and other serious adverse events in a phase 1 study (114). However, after nearly 18 months of investigations, both the U.S. Food and Drug Administration and the European Medicines Agency concluded that these adverse events were not mechanism related but rather resulted from off-target toxicity specific to BIA 10–2474. No similar adverse events have been seen in the hundreds of human research participants who have received other FAAH inhibitors, such as PF-04457845 or JNJ-42165279 (115).

The FAAH inhibitor JNJ-42165279 (101) is currently being evaluated for the treatment of PTSD in a 12-week randomized controlled trial of 90 individuals with PTSD that combines evaluation of established clinical end points with neuroimaging and psychophysiology-based experimental biomarkers, including fear extinction and functional magnetic resonance imaging measures of vmPFC–amygdala connectivity (EudraCT 2020–001965-36). The ongoing PTSD study capitalizes on insights from a recent study where JNJ-42165279 was evaluated for social anxiety, which used a dose of 25 mg once daily of JNJ-42165279, and found only modest effects on self-reported ratings of social anxiety. However, a greater reduction in symptoms was seen in individuals with more complete inhibition of FAAH and higher AEA levels (116). These findings, together with the robust effects observed using the potent, irreversible FAAH inhibitor PF-04457845, highlight a likely need to sustain complete or near-complete FAAH inhibition throughout the 24-hour cycle. In the ongoing PTSD study with JNJ-42165279, a dose of 25 mg twice daily is used to achieve this objective, based on pharmacokinetic and pharmacodynamic modeling. A recent clinical trial reported the efficacy of PF-04457845 as a treatment for cannabis use disorder (117), which could be particularly valuable in the treatment of comorbid PTSD and cannabis use disorder.

### Clinical Trials: MAGL Inhibition

An experimental medicine crossover study in 30 subjects with PTSD has recently been initiated to investigate the effects of MAGL inhibition on neuroimaging biomarkers in this population (NCT04597450). Results have yet to be reported.

## CONCLUSIONS

The first attempt at targeting the endocannabinoid system, via the CB_1_ inverse agonist rimonabant, was infamously ended when the drug was pulled off the shelves by the European Medicines Agency. This was due to adverse events consistent with the current model: blockade of endocannabinoid transmission was associated with mood and anxiety symptoms, including suicidal ideation (118). Although rimonabant had robust efficacy for its approved indication, obesity in type 2 diabetes, these psychiatric symptoms prevented its clinical use. They also supported the notion that enhancing, as opposed to suppressing, cannabinoid signaling may be desirable in mood and anxiety disorders. More effective strategies to exploit the eCB system have since been identified, and the tools to target them are now becoming available for use in humans. Preclinical research has provided overwhelming support for the use of FAAH inhibitors to facilitate the extinction of fear and attenuate anxiety. With proper dosing, THC can produce similar effects. Although support for MAGL inhibitors is less consistent, these could hold promise as well but may critically rely on the identification of the right dosing and level of inhibition.

In contrast to other areas of neuropsychopharmacology, effects of endocannabinoid modulation on the regulation of fear and stress responses have so far translated from preclinical studies to humans. These effects are particularly encouraging in the context of PTSD treatment, as they target the core pathophysiological mechanisms of extinction-resistant fear and exaggerated stress responses, rather than merely mitigating symptoms. A particularly appealing strategy is to combine eCB-potentiating therapeutics with extinction learning in a PE context, thus promoting the consolidation of the extinction memory, while at the same time making the exposure less aversive. Of note, most studies thus far have focused on pharmacological augmentation of extinction learning, although memory reconsolidation processes are also modulated by cannabinoid function and could potentially be explored (119,120). Targeting the endocannabinoid system to treat PTSD could be a long-awaited successful translation of a mechanism identified and behaviorally characterized in animals and brought all the way into the clinic as a novel treatment for PTSD.

## Figures and Tables

**Table 1.** PTSD-Relevant Clinical Trials for Endocannabinoid-Degrading Enzyme Inhibitors

Study Registration	Study Population	Dosing Information	Outcome Measures	Effects of Treatment (vs. Placebo)
FAAH Inhibition: PF-04457845				
EudraCT 2016-005013-47 [Mayo *et al.* (97)]	Healthy adults, *N* = 45	4 mg/day for 10 days	Fear-potentiated startle	
			Conditioned fear extinction	No effect
			Recall of extinction (24 hours later)	Greater
			Stress	
			Skin conductance response	Reduced
			Subjective stress	Reduced
			Negative affect	Reduced
			Cortisol	No effect
NCT01665573 (results not reported)	Healthy adults, *N* =150	Unknown dose, unknown duration	Psychophysiology	
			Conditioned fear extinction	NA
			Stress	
			Cortisol	NA
NCT02216097 (premature termination)	PTSD, *N* = 14	4 mg/day for 7 days	Neuroimaging	
			Emotional faces	NA
			Fear extinction	NA
			Fear extinction retention	NA
EudraCT 2014-002456-90 (premature termination)	Comorbid PTSD and AUD, *N* = 150, women only	4 mg/day for 4 weeks + exposure therapy	Clinical questionnaires	
			Clinician-administered PTSD Scale	NA
			Alcohol consumption (TLFB)	NA
			Stress	
			Hair cortisol	NA
FAAH Inhibition: JNJ-42165279				
NCT01826786 [Paulus et *al.* (98)]	Healthy adults, *N* = 45, men only	100 mg/day for 4 days	Neuroimaging	
			Emotional face processing	Reduced (amygdala, ACC, insula)
			Breathing restriction	Increased (ACC, anterior insula)
			Fear conditioning and extinction	No effect
NCT02432703 [Schmidt *et al.* (116)]	Social anxiety disorder, *N* = 149	25 mg/day for 12 weeks	Clinical questionnaires	
			Liebowitz Social Anxiety Scale	No effect^a^
			Hamilton Anxiety Ratings Scale	Significant decrease
			Hamilton Depression Rating Scale	No change (low to start with)
			Clinical Global Impression Scale	Greater improvement
			Plasma measures	
			Drug levels, anandamide	Higher dose may be needed^a^
NCT02498392 (results not yet reported)	MDD + anxious distress, *N* =161	25 mg/day for 6 weeks + SSRI	Clinical questionnaires	
			Hamilton Depression Rating Scale	TBD
			Hamilton Anxiety Rating Scale	TBD
			Plasma drug concentrations	TBD
EudraCT 2020-001965-36 (ongoing)	PTSD (*N* = 90), placebo-controlled, blinded	25 mg × 2/day, 12 weeks + exposure therapy	Clinical questionnaires	
			Clinician-administered PTSD Scale	TBD
			PTSD Checklist	TBD
			Pittsburgh Sleep Quality Index	TBD
			Neuroimaging	
			Emotion conflict	TBD
			Emotion regulation	TBD
			Fear-potentiated startle	
			Conditioned fear extinction	TBD
			Extinction recall	TBD
			Stress reactivity	
			Skin conductance response	TBD
			Subjective stress ratings	TBD
			Cortisol	TBD
MAGL Inhibition: Lu AG06446				
NCT04597450 (ongoing)	PTSD (*N* = 30), placebo-controlled, blinded	Not reported	Neuroimaging	
			Facial affect recognition	TBD
			Threat processing	TBD
			Card guessing task	TBD
			Sleep polysomnography	TBD

Ongoing, completed, or terminated studies using PTSD-relevant measures (e.g., fear, stress) or populations involving inhibition of FAAH or MAGL inhibition to augment anandamide or 2-AG, respectively.

ACC, anterior cingulate cortex; AUD, alcohol use disorder; FAAH, fatty acid amide hydrolase; MAGL, monoacylglycerol lipase; MDD, major depressive disorder; NA, not available; PTSD, posttraumatic stress disorder; SSRI, selective serotonin reuptake inhibitor; TBD, to be determined; TLFB, timeline follow back.

a Individuals with higher anandamide levels had greater improvements in social anxiety; plasma analysis revealed incomplete FAAH inhibition, suggesting that a higher dose may be needed.
